# Knowledge and Attitudes of Medical Students and Interns with Regard to Female Feticide

**DOI:** 10.4103/0970-0218.51217

**Published:** 2009-04

**Authors:** Anita Nath, Nandini Sharma, Gopal K. Ingle

**Affiliations:** Population Council, 1230 York Avenue, New York City, New York-10065, United States; 1 Department of Community Medicine, Maulana Azad Medical College, Bahadur Shah Zafar Marg, New Delhi - 110 002, India. E-mail: anath@popcouncil.org

Sir,

According to a recent report by UNICEF, up to 50 million girls and women are missing from India's population as a result of systematic gender discrimination.(1) The 2001 Census reported an adverse child sex ratio of 927.(2) The proliferation and abuse of advanced technology coupled with the low status of women has made the evil practice of female feticide common in middle and higher socio-economic households, especially in the Northern states.(3,4) However, in contrast to these observations, another study reveals that the age-old son-preference has slightly declined at the end of the twentieth century, and a substantial selective male feticide is also being committed annually along with larger selective female feticide.(5) If the doctors are willing to fight against this emerging concern, the problem could easily be curbed.

This study was conducted to assess the perspectives of tomorrow's doctors on this issue. Study participants included 62 interns and 39 MBBS students in their seventh semester who were posted to the Department of Community Medicine of Maulana Azad Medical College, New Delhi. They were asked to complete a pre-designed and pre-tested questionnaire that contained multiple choice questions pertaining to their knowledge and attitude toward female feticide. The data was entered using SPSS Version 13.0 software and analyzed by means of comparison of simple proportions.

Out of 100 medical undergraduates, 57% were males and 43% were females. The mean age of students was 21.8 ± 0.6 years while the mean age of interns was 23.2 ± 0.8 years. Table 1 displays the participants' responses regarding the danger of female feticide and possible measures to curb this practice. It is important to note that less than a third of the participants were in favor of stricter punishment for the doctors involved in this practice. A significantly higher number of female participants were in favor of woman empowerment related strategies.

**Table 1:** The dangers of female feticide and suggestions pertaining to steps to arrest the declining gender ratio

	Numbers (%)		
	
	Male (n=57)	Female (n=43)	Total (n=100)
Dangers*			
Increasing sexual and social crimes against women	36 (63.2)	28 (65.1)	64 (64)
Increase in prostitution, sexual exploitation and cases of sexually transmitted infection and HIV/AIDS**	31 (54.4)	35 (81.4)	66 (66)
Effect on women's health because of repeated pregnancies and forced abortion	30 (52.6)	23 (53.4)	53 (53)
Suggestions*			
Stricter punishment for doctors conducting illegal			
Medical termination of pregnancy	10 (17.5)	5 (11.6)	15 (15)
Stricter punishment for doctor conducting illegal ultrasounds	15 (26.3)	11 (25.6)	26 (26)
Stricter punishment for woman seeking abortions	9 (15.7)	5 (11.6)	14 (14)
Stricter punishment for woman's family**	28 (49.2)	28 (65.2)	56 (56)
Raising woman's status in society**	40 (70.2)	38 (88.3)	78 (78)
Improving employment opportunities for women**	22 (38.6)	26 (60.4)	48 (48)
Support the cause of girl child by means of mass media	36 (63.2)	29 (67.4)	65 (65)

* Includes multiple responses,

** Indicates statistical significance: (*P*<0.05)

The findings in our study underscore the need to sensitize tomorrow's doctors about the ethics related to the inappropriate and indiscriminate use of technology. This could be brought about by conducting regular workshops/Continuing Medical Education sessions (CMEs) and awareness campaigns in field practice areas of the department. Private practitioners should also be encouraged to participate in such programs. While the future doctors could join hands in efforts directed at improving the status of women in India, it is more urgent that they unite in curbing the threat posed by doctors involved in such practices and, most importantly, be sensitized enough so as to not get involved themselves.
